# Correlation between structural heart disease and cardiac SARS-CoV-2 manifestations

**DOI:** 10.1038/s43856-022-00204-6

**Published:** 2022-11-11

**Authors:** Felix Nägele, Michael Graber, Jakob Hirsch, Leo Pölzl, Sabina Sahanic, Manuel Fiegl, Dominik Hau, Clemens Engler, Sophia Lechner, Anna Katharina Stalder, Kirsten D. Mertz, Jasmin D. Haslbauer, Alexandar Tzankov, Michael Grimm, Ivan Tancevski, Johannes Holfeld, Can Gollmann-Tepeköylü

**Affiliations:** 1 Department of Cardiac Surgery, Medical University of Innsbruck, Innsbruck, Austria; 2 Department of Internal Medicine II, Medical University of Innsbruck, Innsbruck, Austria; 3 Department of Internal Medicine I, Medical University of Innsbruck, Innsbruck, Austria; 4 Institute of Medical Genetics and Pathology, University Hospital Basel, Basel, Switzerland; 5 Institute of Pathology, Cantonal Hospital Baselland, Liestal, Switzerland

**Keywords:** Cardiomyopathies, Inflammation

## Abstract

**Background:**

The prognosis of COVID-19 patients with cardiac involvement is unfavorable and it remains unknown which patients are at risk. The virus enters cells via its receptor angiotensin-converting enzyme 2 (ACE2). Myocardial ACE2 expression is increased in structural heart disease (SHD). We, therefore, aimed to analyze correlations between structural heart disease and cardiac SARS-CoV-2 manifestation.

**Methods:**

The clinical course of COVID-19 in patients with structural heart disease was assessed in a prospective cohort of 152 patients. The primary endpoints consisted of hospitalization and survival. Cardiac tissue of 23 autopsy cases with lethal COVID-19 course was obtained and analyzed for (a) the presence of SHD, (b) myocardial presence of SARS-CoV-2 via RT,-PCR, and (c) levels of ACE2 expression using immunofluorescence staining.

**Results:**

Structural heart disease is found in 67 patients, of whom 56 (83.60%) are hospitalized. The myocardium is positive for SARS-CoV-2 in 15 patients (65%) in 23 autopsy cases of lethal COVID-19. Moreover, most hearts with evidence of myocardial SARS-CoV-2 have structural heart disease [11 (91,67%) vs. 1 (8,33%), *p* = 0.029]. Myocardial presence of SARS-CoV-2 is correlated with a significant downregulation of ACE2 compared to negative control hearts (6.545 ± 1.1818 A.U. vs. 7.764 ± 2.411 A.U., *p* = 0.003). The clinical course of patients with cardiac SARS-CoV-2 manifestation is unfavorable, resulting in impaired survival (median, 12 days and 4.5 days, respectively, HR 0.30, 95% CI, 0.13 to 0.73, *p* = 0.0005)

**Conclusions:**

We provide evidence for a correlation between SHD, altered ACE2 receptor expression, and cardiac SARS-CoV-2 manifestation. Consequently, structural heart disease may be considered a distinct risk factor for a severe clinical course after infection with SARS-CoV-2.

**Registration number local IRB:**

Ethics Committee of Northwestern and Central Switzerland ID 2020-00629; Ethics Committee of the Medical University Innsbruck EK Nr: 1103/2020.

**ClinicalTrials.gov number:**

NCT04416100.

## Plain language summary

SARS-CoV-2, the virus that causes COVID-19, binds to ACE2 receptors to gain entry into cells. The ACE2 receptor is a cell surface protein found in many tissues, including the heart. Studies suggest that people with heart disease are likely to have higher levels of ACE2 receptors, which may explain why they are more susceptible to severe illness from COVID-19. In this study, we identified heart disease as a risk factor for hospitalization in 152 patients who tested positive for SARS-CoV-2. The presence of SARS-CoV-2 in the heart was associated with altered levels of ACE2 receptors and with a shortened survival time in patients. These findings provide evidence for a potential link between heart disease, ACE2 receptor levels, and SARS-CoV-2 infection of the heart, and may help doctors to understand the clinical course of patients with heart disease who contract COVID-19.

## Abstract (web summary)

Nägele, Graber et al. evaluate cardiac manifestations of SARS-CoV-2 in patients with structural heart disease. The authors find that detection of SARS-CoV-2 in heart tissue is associated with poorer survival.

## Introduction

The coronavirus disease 19 (COVID-19) caused by SARS-CoV-2 infections remains a significant challenge for healthcare systems worldwide^1,2^. Despite intensive research and a plethora of newly published data regarding underlying pathomechanisms of SARS-CoV-2 infections and its clinical course, there is an unmet need to identify specific vulnerable groups prone to severe course of the disease by the affection of organs other than the lungs^3^. The viral spike protein infects cells via the angiotensin converting enzyme type 2 (ACE2) that serves as a receptor; ACE is a crucial mediator of the renin-angiotensin system^4–6^.

As the most prevalent cell types in the heart – namely cardiomyocytes, fibroblasts, and endothelial cells—express ACE2, SARS-CoV-2 infection may be accompanied by cardiovascular involvement^7^.

Infection of endothelial cells may result in endothelial dysfunction with prothrombotic events resulting in myocardial infarction or Takotsubo syndrome^8^. In vitro studies suggest possible infection of cardiomyocytes with SARS-CoV-2 causing cell death and dysfunction indicated by elevated serum levels of troponin T and NT-proBNP and impact on the cardiac conduction system^8^. Infection of fibroblasts might promote cardiac remodeling resulting in fibrosis and impairment of cardiac function^9^.

COVID-19 patients with cardiac involvement exhibit an aggravated clinical course with poor prognosis, including higher morbidity and mortality^10^. It remains unclear whether these symptoms occur due to (a) viral infection of the myocardium or (b) secondary myocardial injury due to a fierce cytokine storm induced by the innate immune system in response to the infection^11^. Despite increasing evidence regarding cardiac involvement in COVID-19, it remains unclear which patients are at high risk for cardiac involvement. ACE2 expression in cardiomyocytes is increased in patients with structural heart disease, particularly in patients with cardiac hypertrophy^4^. Expression levels of this receptor crucial for cellular SARS-CoV 2 infection might determine cardiac involvement in COVID-19^4,5,12^.

In this study, we provide evidence for a correlation between SHD, altered ACE2 receptor expression, and cardiac SARS-CoV-2 manifestation. These findings suggest that structural heart disease may be considered a distinct risk factor for cardiac involvement upon SARS-CoV2 infections, potentially leading to a severe clinical course.

## Methods

### CovILD cohort

Patient recruitment and data collection were performed within the CovILD PLUS Study (ClinicalTrials.gov number, NCT04416100), a prospective, multi-center, observational cohort trial as described previously^12^. Enrolment of patients was initiated on April 29th, 2020, at the Department of Internal Medicine II, Medical University of Innsbruck (Austria), with two additional study sites: St. Vinzenz Hospital Zams and Rehabilitation Facility Münster (both located in Austria). Patients were asked to attend study visits six and twelve weeks after disease onset. All included study participants had a clinical COVID-19 presentation and laboratory-confirmed SARS-CoV-2 infection according to the WHO guidelines. In total, 190 patients were screened for study participation, and 152 were prospectively included. Written informed consent was obtained from each study participant. The study protocol was granted approval by the local ethics committee at the Medical University of Innsbruck (EK Nr: 1103/2020) and was conducted in accordance with the declaration of Helsinki. All study participants underwent clinical examination, laboratory testing, and echocardiography performed according to the recommendations of the European Society of Cardiology^13^. Structural heart disease was defined as a collective finding of ventricular hypertrophy and/or sclerotic or dysfunctional aortic/mitral valve as indicative of myocyte hypertrophy initiation.

### Autopsy cases cohort

Autopsies of twenty-three COVID-19 fatalities, diagnosed as per ante-mortem nasopharyngeal swab, were performed from March to June 2020 at the Institutes of Pathology of the University Hospital of Basel (*n* = 12) and the Cantonal Hospital Baselland, Liestal (*n* = 11). A full-body autopsy was performed in 21 cases (91%). In cases of excessive overweight or according to patient or relatives’ wishes, a partial autopsy of the upper respiratory tract, lungs, and heart (*n* = 2, 9%) was conducted. COVID-19-associated respiratory failure was the cause of death in all 23 cases. Respiratory failure was due to either acute respiratory distress syndrome/diffuse alveolar damage (ARDS/DAD), and/or pulmonary embolism (PE), and/or bronchopneumonia. Subgroups were defined by myocardial reverse transcription polymerase chain reaction (RT-PCR) cycle threshold (CT) values, and 15 hearts (65%) of the 23 COVID-19 fatalities appeared to be SARS-CoV-2 positive^14^. Cardiac expression levels of ACE2 were determined by immunofluorescence staining. The use of samples and data obtained from the autopsy cohort was granted approval by the local Ethics Committee of Northwestern and Central Switzerland (ID 2020-00629) and was conducted in accordance with the declaration of Helsinki. Written informed consent for the use of the patient’s samples in research was obtained before the autopsy by relatives if the patient did not already give written informed consent.

### Histopathology

A single-blinded pathologist performed all histological examinations. Tissue sections (0.4 × 1 × 2 cm) of the left ventricle and interventricular septum were collected at autopsy and subsequently fixed in 4% buffered formalin, paraffin-embedded, stained with Hematoxylin/Eosin (H&E) and assessed by light microscopy.

### SARS-CoV-2 detection and RT-PCR

The Maxwell RSC RNA FFPE Kit (Promega, Madison, WI, USA) was used to extract RNA from formalin-fixed paraffin-embedded myocardium (FFPE) as described previously^15^. A comparative cycle threshold (ΔΔCт) method using the TaqMan 2019-nCoV Control Kit v1 (Thermo Fisher Scientific) was utilized to quantify genome copy number, generating separate copy numbers for three different viral genomic regions [ORFab1 (open reading frame), S (spike) and N (nucleocapsid)] and the human *RPPH1* gene (RNAse-P) to determine cardiac SARS-CoV2 infection. According to the manufacturer’s protocol, a Cт value below 37 in any viral genomic region was considered positive. A case was deemed negative if all Cт values were above 40. Values between 37 and 40 were considered undetermined, and the assay was repeated. Samples were run in duplicates. For correlation analysis, RNA expression levels of ACE2, TNFa, IFNa, and SARS-CoV-2 spike protein were determined with the following primer:

ACE2 FWD: ggg atc aga gat cgg aag aag aaa

ACE2 REV: agg agg tct gaa cat cat cag tg

IL6 FWD: agc cac tca cct ctt cag aac

IL6 REV: agt gcc tct ttg ctg ctt tc

IFNa FWD: atg gtc ctg gtg gtg gtc agc t

IFNa REV: atc cag gct gtg ggt ctc agg g

spike protein FWD: acg gcc tta ctg ttt tgc cac ct

spike protein REV: cag cac ctg cac caa agg tcc a

### Immunofluorescence

For immunofluorescence staining, sections underwent deparaffination and subsequently a heat-mediated antigen retrieval in sodium-citrate buffer (10 mM sodium-citrate, 0.05% Tween 20, pH 6,0). Upon washing with PBS and incubation with blocking buffer (3% BSA, 0.1% TritonX, 0.05% Tween 20), slides were incubated with primary antibodies anti-ACE2 (ab15348, Abcam) and anti-alpha smooth muscle actin (ab5694, Abcam) overnight. AlexaFluor488 (A11001, Invitrogen) and AlexaFluor568 (A11011, Invitrogen) were used as secondary antibodies. For counterstaining, DAPI was used (62248, Thermo Scientific). Sections were z-stack imaged with a LSM980 confocal microscope (Carl Zeiss, Germany). Subsequent 3D rendering was performed using the Imaris software Version 9.6 (Bitplane AG, Switzerland). Images were analyzed automatedly as described previously ^16^.

### Statistical analysis

Data are presented as mean ± SD for continuous variables, absolute numbers, and percentages for categorical variables. The two groups (either hypertrophic or non-hypertrophic and ACE2 positive or negative, respectively) were compared for differences in demographic patient characteristics outcomes. As appropriate, comparisons between two groups were performed for categorical variables with the Chi-Square or Fisher’s exact test. Correlation analysis was performed with Spearman’s rank correlation. Continuous variables were compared by Student’s t-test or Mann–Whitney U test. Data documentation and statistical analysis were performed using SPSS 24.0 (IBM Corp.) and RStudio Version 1.4 (RSudio Team, Boston, USA).

### Reporting summary

Further information on research design is available in the Nature Research Reporting Summary linked to this article.

## Results

**Fig. 1:**
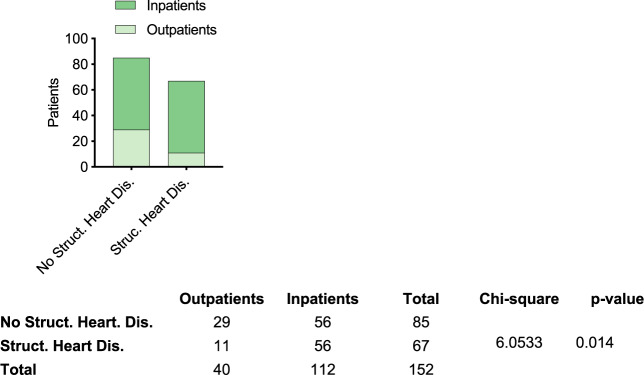
Relationship between structural heart disease and hospitalization rates upon SARS-CoV-2 infection. A significant relationship between pathological echo findings and hospitalization rate in 152 patients who tested positive for SARS-CoV-2 could be found (*p* < 0.0002, Chi-square test).

First, we aimed to clarify whether patients with structural heart disease indeed had a more severe COVID-19 clinical course. For this purpose, we conducted echocardiography studies in a cohort of 152 patients tested positive for SARS-CoV-2. Structural heart disease, defined as echocardiographic abnormalities including valvular pathologies and ventricular hypertrophy, could be found in 67 patients, of whom 56 (83.60%) had to be hospitalized. In contrast, hospitalization rate was markedly lower in patients without structural heart disease (27.5%, *p* = 0.023) (Fig. 1). Inpatients were older (59.5 [53.0;71.2] years vs. 47.5 [36.8;55.0] years, *p* < 0.001), more often male [73 (65.2%) vs. 13 (32.5%), *p* = 0.001] and had a higher BMI [26.1 (24.2;29.3) vs. 24.8 (20.9;27.6), *p* = 0.038] than patients who were not hospitalized. Moreover, patients with diabetes [23 (20.9%) vs. 1 (2.86%), *p* = 0.025], hypertension [42 (38.2%) vs. 2 (5.71%), *p* = 0.001] or hyperlipidemia [25 (22.7%) vs. 2 (5.71%), *p* = 0.045] had to be hospitalized more often. Fever [86(78.2%) vs. 19(55.9%), *p* = 0.019] and weight loss [87(79.1%) vs. 14 (41.2%), *p* = 0.019] were more common symptoms among hospitalized patients, while pain was more often reported by outpatients [26 (76.5%) vs. 52 (47.3%)]. TroponinT (8.20 [5.30;13.5] pg/ml vs. 5.00 [5.00;5.00] pg/ml, *p* < 0.001), Neurophils (3.70 [2.73;4.57] g/L vs. 3.10 [2.65;3.70] g/l, *p* = 0.034) and LDH (203 [183;223] U/L vs. 176 [148;204] U/L, *p* < 0.001) levels were significantly higher in hospitalized patients (Table 1).

**Table 1:** Clinical characteristics such as demographics, comorbidities, medication, and laboratory results of the CovILD Cohort.

	ALL	Outpatients	Inpatients	*p*-value
	*N* = 152	*N* = 40	*N* = 112	
Sex, male	86 (56.6%)	13 (32.5%)	73 (65.2%)	0.001
BMI, median [IQR]	25.8 [23.4;28.7]	24.8 [20.9;27.6]	26.1 [24.2;29.3]	0.038
Age, years (SD)	56.0 [49.0;69.0]	47.5 [36.8;55.0]	59.5 [53.0;71.2]	<0.001
Follow-up, days [IQR]	128 [117;140]	133 [106;144]	128 [118;139]	0.662
Hospitalization, days [IQR]	7.00 [0.00;15.0]	0.00 [0.00;0.00]	10.0 [6.00;20.2]	<0.001
Oxygen supply	71 (48.6%)	1 (2.63%)	70 (64.8%)	<0.001
ICU admission	33 (22.0%)	0 (0.00%)	33 (29.7%)	<0.001
Diabetes	24 (16.6%)	1 (2.86%)	23 (20.9%)	0.025
Cardiovascular disease	58 (40.0%)	2 (5.71%)	56 (50.9%)	<0.001
Structural heart disease	67 (44.1%)	11 (27.5%)	56 (50.0%)	0.023
Hypertension	44 (30.3%)	2 (5.71%)	42 (38.2%)	0.001
Hyperlipidemia	27 (18.6%)	2 (5.71%)	25 (22.7%)	0.045
COPD	8 (5.52%)	1 (2.86%)	7 (6.36%)	0.680
Infectious lung disease	1 (0.69%)	0 (0.00%)	1 (0.91%)	1.000
Asthma	10 (6.90%)	3 (8.57%)	7 (6.36%)	0.704
COVID SYMPTOMS				
Symptom onset, days [IQR]				
Dyspnea, NYHA [IQR]	1.00 [0.00;4.00]	1.00 [0.00;3.00]	1.00 [1.00;4.00]	0.186
Cough	101 (70.1%)	22 (64.7%)	79 (71.8%)	0.564
Fever	105 (72.9%)	19 (55.9%)	86 (78.2%)	0.019
Night sweat	91 (63.2%)	20 (58.8%)	71 (64.5%)	0.688
Weightloss	101 (70.1%)	14 (41.2%)	87 (79.1%)	<0.001
pain	78 (54.2%)	26 (76.5%)	52 (47.3%)	0.005
GI symptoms	59 (41.0%)	14 (41.2%)	45 (40.9%)	1.000
Anosmia	61 (42.4%)	16 (47.1%)	45 (40.9%)	0.663
Sleep disorders	56 (39.4%)	17 (50.0%)	39 (36.1%)	0.214
Dermatologic symptoms	25 (17.4%)	9 (26.5%)	16 (14.5%)	0.178
LAB RESULTS				
NT-pro-BNP	84.5 [50.0;210]	79.0 [50.0;144]	86.0 [50.0;278]	0.066
sACE [IQR]	35.0 [26.0;46.0]	34.5 [26.8;39.5]	36.0 [26.0;48.0]	0.400
TropT [IQR]	6.20 [5.00;11.0]	5.00 [5.00;5.00]	8.20 [5.30;13.5]	<0.001
WBC (10^9/L) [IQR]	6.00 [5.07;7.03]	5.85 [5.00;6.25]	6.15 [5.10;7.20]	0.126
Neutrophils (g/L) [IQR]	3.50 [2.70;4.23]	3.10 [2.65;3.70]	3.70 [2.73;4.57]	0.034
Hemoglobin [g/L] [IQR]	137 [129;148]	135 [128;143]	138 [130;149]	0.316
Thrombocytes [10^9/L] [IQR]	252 [217;295]	253 [226;288]	252 [214;296]	0.573
% segmented Neutrophils, mean (SD)	58.7 (10.1)	55.8 (9.12)	59.5 (10.2)	0.048
% Lymphocytes, mean (SD)	30.3 (9.13)	33.7 (8.74)	29.3 (9.04)	0.013
Lymphocytes (10^9/L) [IQR]	1.75 [1.41;2.12]	1.85 [1.60;2.33]	1.73 [1.40;2.09]	0.236
Creatinin (mg/dL) [IQR]	0.81 [0.70;0.95]	0.78 [0.71;0.85]	0.83 [0.70;0.96]	0.295
LDH (U/L) [IQR]	198 [174;218]	176 [148;204]	203 [183;223]	<0.001
INR [IQR]	0.90 [0.90;1.00]	0.90 [0.90;1.00]	0.90 [0.90;1.00]	0.461
CRP (mg/dL) [IQR]	0.15 [0.06;0.33]	0.10 [0.06;0.26]	0.15 [0.07;0.36]	0.165

Continuous data are presented as median (interquartile range) in cases of non-normal distribution or mean ± standard deviation in cases of normal distribution; categorical data are presented as *n* (%).

**Fig. 2:**
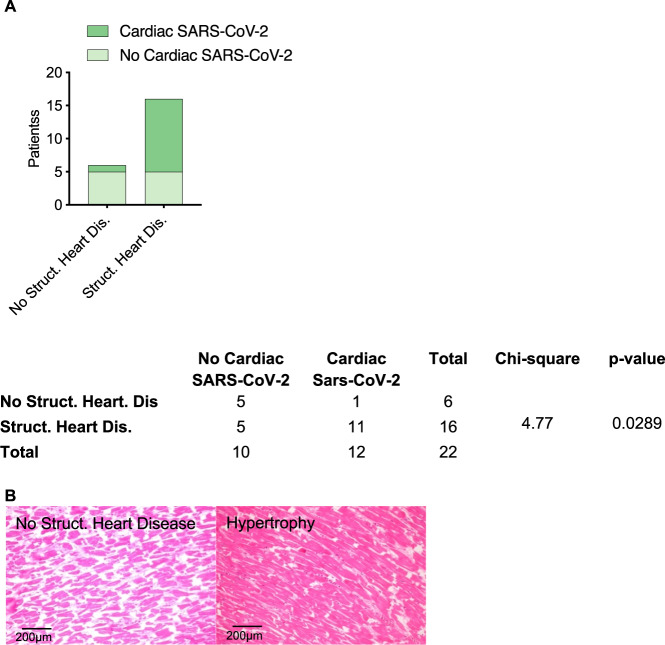
Histopathological signs of cardiac hypertrophy in correlation to SARS-CoV-2 infections. **A** Patients suffering from structural heart disease showed higher rates of cardiac SARS-CoV-2 infections. (*p* = 0.0289, Chi-square test). **B** Representative histopathological images of Hematoxylin/Eosin stained tissue sections from hypertrophic and non-hypertrophic hearts.

To verify whether patients with structural heart disease indeed showed higher rates of cardiac SARS-CoV-2 infections, we examined hearts of 23 autopsy cases of lethal COVID-19. Heart tissue was positive for SARS-CoV-2 in 15 patients (65%). These patients were more often male [4 (50.0%) vs. 14 (93.3%), *p* = 0.033], died earlier after hospitalization (12.0 days [9.00;15.2] vs. 4.00 days [3.00;6.00], *p* = 0.001), had less often fever [8 (100%) vs. 7 (46.7%), *p* = 0.019] but more often suffered from impaired renal function (Creatinin 68.0 [49.5;98.5] vs. 242 [123;450], *p* = 0.030) (Table 2). Indeed, most hearts with evidence of cardiac SARS-CoV-2 infection showed signs of cardiac hypertrophy [11 (91,67%) vs. 1 (8,33%), *p* = 0.029)] (Fig. 2).

**Table 2:** Clinical characteristics such as demographics, comorbidities, medication, and laboratory results of 23 autopsy cases with lethal COVID-19 course.

	All	No Cardiac SARS-CoV-2	Cardiac SARS-CoV-2	*p*-value
	*N* = 23	*N* = 8	*N* = 15	
Sex, male	18 (78.3%)	4 (50.0%)	14 (93.3%)	0.033
Age, years (SD)	75.6 (12.5)	69.9 (13.3)	78.6 (11.4)	0.141
BMI, median [IQR]	27.0 [26.0;32.5]	28.5 [26.5;36.5]	27.0 [26.0;29.0]	0.475
Hospitalization, days [IQR]	7.00 [3.50;10.5]	12.0 [9.00;15.2]	4.00 [3.00;6.00]	0.001
Cough	4 (17.4%)	0 (0.00%)	4 (26.7%)	
Dyspnea	10 (43.5%)	3 (37.5%)	7 (46.7%)	1.000
Fever	15 (65.2%)	8 (100%)	7 (46.7%)	0.019
Hypertension	20 (87.0%)	7 (87.5%)	13 (86.7%)	1.000
Cardiovascular disease	17 (73.9%)	6 (75.0%)	11 (73.3%)	1.000
COPD	11 (47.8%)	3 (37.5%)	8 (53.3%)	0.667
DM Type 2	9 (39.1%)	4 (50.0%)	5 (33.3%)	0.657
MEDICATION				
RAAS Inh. before admission	13 (56.5%)	3 (37.5%)	10 (66.7%)	0.221
Anticoagulation and/or platelet aggregation inhibition before admission	15 (65.2%)	5 (62.5%)	10 (66.7%)	1.000
Anticoagulation and/or platelet aggregation inhibition during hospitalization	21 (91.3%)	7 (87.5%)	14 (93.3%)	1.000
hydroxychloroquine during hospitalization	15 (65.2%)	3 (37.5%)	12 (80.0%)	0.071
tocilicumab during hospitalization	5 (21.7%)	0 (0.00%)	5 (33.3%)	0.122
LAB RESULTS				
CRP (mg/dL) [IQR]	199 (120)	170 (98.3)	215 (131)	0.402
Leucocytes, median [IQR]	8.79 [7.10;11.9]	7.80 [6.70;8.60]	9.41 [7.48;15.0]	0.247
Lymphocytes, median [IQR]	0.61 [0.40;0.96]	0.96 [0.65;1.29]	0.50 [0.40;0.70]	0.156
Neutrophils, median [IQR]	6.80 [4.40;10.1]	6.05 [3.85;6.78]	7.35 [6.64;10.5]	0.086
Hemoglobin, median [IQR]	109 [96.0;126]	112 [97.5;116]	108 [95.5;128]	0.794
Erythrocytes, median [IQR]	3.54 (1.27)	2.85 (0.72)	3.68 (1.33)	0.309
Thrombocytes, median [IQR]	126 [92.0;225]	104 [81.5;264]	143 [106;213]	0.765
INR, median [IQR]	1.30 [1.20;1.58]	1.25 [1.20;1.37]	1.30 [1.20;1.75]	0.599
aPPT, median [IQR]	38.0 [34.5;68.5]	50.0 [43.0;57.0]	38.0 [33.0;73.0]	1.000
D-Dimer, median [IQR]	4.58 [1.80;6.57]	6.93 [6.93;6.93]	3.69 [1.17;5.48]	0.380
LDH, median [IQR]	628 [343;753]	663 [654;752]	444 [321;753]	0.463
Creatinin, median [IQR]	133 [72.0;407]	68.0 [49.5;98.5]	242 [123;450]	0.030
CK-MB, median [IQR]	5.40 [4.00;20.8]	31.2 [31.2;31.2]	5.05 [3.65;9.08]	0.317
hsTropT, median [IQR]	109 (90.1)	80.6 (56.1)	126 (108)	0.467

Angiotensin-converting enzyme inhibitors, angiotensin receptor blockers, renin inhibitors, and aldosterone inhibitors were defined as renin-angiotensin-aldosterone system (RAAS) inhibitors. Anticoagulants included: heparin and its derivatives, new oral anticoagulants, coumarin derivatives, and/or platelet aggregation inhibitors. Continuous data are presented as median (interquartile range) in cases of non-normal distribution or mean ± standard deviation in cases of the normal distribution; categorical data are presented as *n* (%).

**Fig. 3:**
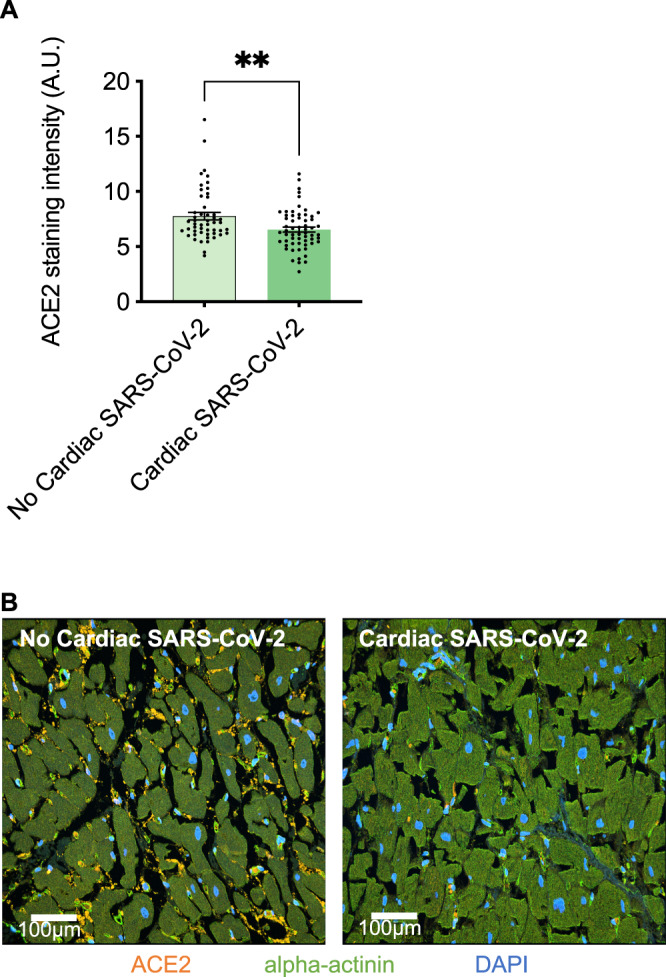
Altered ACE2 expression upon cardiac SARS-CoV-2 infection. **A** Cardiac ACE2 staining intensity was significantly downregulated in hearts with cardiac SARS-CoV-2 viral load (*p* = 0.003, Unpaired t-test, *n* = 5 high-power fields per section). **B** Representative immunofluorescence images of ACE2 and alpha-actinin stained tissue sections from hearts without and with cardiac SARS-CoV-2 infections.

**Fig. 4:**
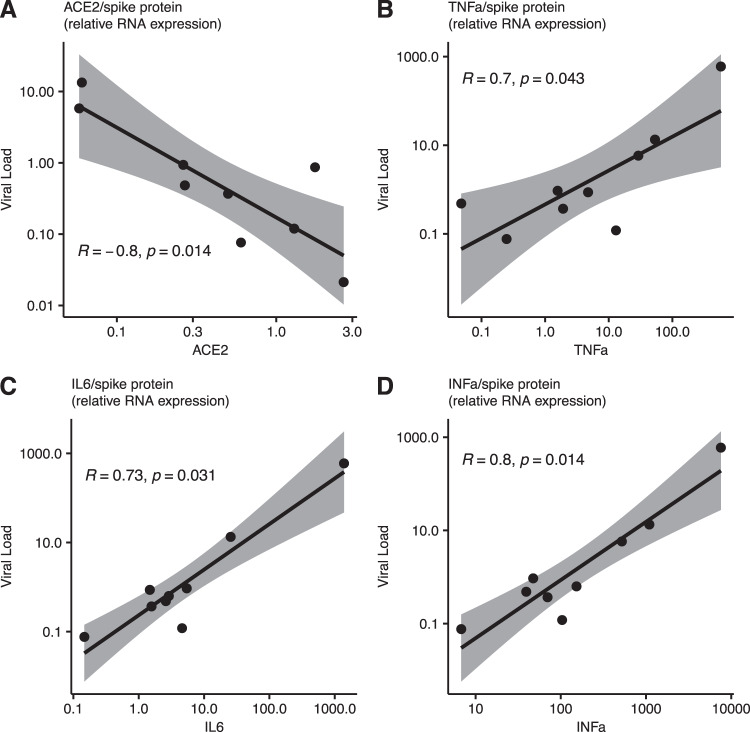
Cardiac ACE2 and cytokine expression levels correlate with cardiac viral load. **A** Increased SARS-CoV-2 spike protein RNA expression is significantly correlated with reduced gene expression levels of ACE2 and increased cytokine expression levels of **B** TNFa, **C** IL6, and **D** IFNa (Spearman’s rank correlation, regression slopes are represented by black lines, grey area represents 95% CI, *n* = 9).

**Fig. 5:**
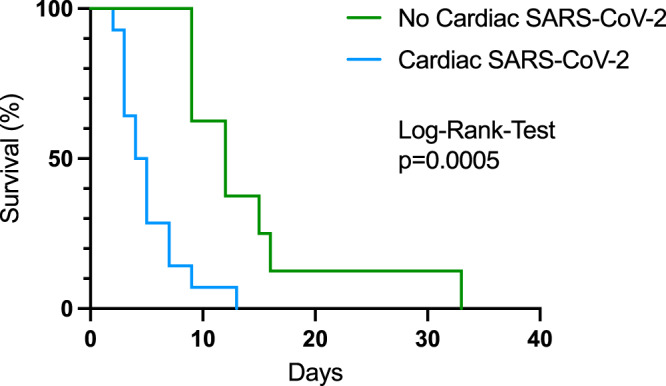
Short-term survival of 23 COVID-19 fatalities. Cardiac SARS-CoV-2 infection (blue line, *n* = 12) correlated with significantly shorter survival (Log-Rank-Test *p* = 0.005) compared to patients without cardiac viral load (green line, *n* = 10).

To investigate the potential role of myocardial ACE2 expression with cardiac SARS-CoV-2 infections, hearts were stained via immunofluorescence staining for ACE2 expression. Indeed, cardiac infection was accompanied by a significant downregulation of ACE2 compared to negative control hearts (Fig. 3). In line with this, downregulation of cardiac mRNA expression levels was significantly correlated with cardiac viral load (Fig. 4A), while increased cytokine expression levels of tumor necrosis factor alpha (TNFa), interleukin 6 (IL6), and interferon-alpha (IFNa) could be positively correlated to increased viral RNA expression (Fig. 4B–D). Finally, the clinical course of patients with cardiac SARS-CoV-2 infection was unfavorable, resulting in impaired survival (median, 12 days and 4.5 days, respectively, hazard ratio 0.30, 95% CI, 0.13 to 0.73, *p* = 0.0005) (Fig. 5).

## Discussion

COVID-19 remains a global health crisis. Clinical manifestations of COVID-19 mainly consist of fever, dry cough, and dyspnoea^17^. However, 14% of infected patients develop a severe clinical course with the need for invasive respiratory support, intensive care, and 2.3% even die^18^. Patients with cardiac involvement after SARS-CoV 2 infection have an unfavorable prognosis with poor survival. In a prospective cohort study of 416 patients with COVID-19, cardiac involvement occurred in 19.7% of the patients and was associated with a higher risk of in-hospital mortality^19^. Although age and severe comorbidity, including cancer and immunosuppression, have been identified as risk factors for a severe course of COVID-19, it remains unknown which patients are at risk for cardiac involvement after SARS-CoV-2 infection^3^.

The glycosylated outer membrane spike protein of the SARS-CoV-2 binds to the Angiotensin-converting enzyme 2 (ACE2) receptor, thus infecting cells^20^. ACE2 expression is not limited to the respiratory tract but is highly abundant in the cardiovascular system making it vulnerable to SARS-CoV-2 infections^21^. Patients with structural heart disease exhibit increased expression of ACE2 in cardiomyocytes^4^. Therefore, we hypothesized in this present study that the increased expression of ACE2 in patients with structural heart disease might be associated with (a) cardiac SARS-CoV-2 manifestation and (b) impaired clinical outcome.

In a prospective cohort of 152 patients with confirmed COVID-19, we found that pre-existing structural heart disease was a risk factor for hospitalization. These findings align with previous reports correlating echocardiographic abnormalities with a severe clinical course of COVID-19^22,23^. The observed phenomenon’s underlying cause might be attributed to increased cardiac ACE2 expression in patients with structural heart disease^5,6^. Interestingly, pharmacologic induction of ACE2 expression via ACE-I or ARB is not associated with severe COVID-19^24,25^.

To investigate a possible link between ACE2 expression and myocardial SARS-CoV-2 infection, we obtained cardiac tissue of 23 COVID-19 fatalities and analyzed both ACE2 expression levels and SARS-CoV-2 viral load. Indeed, cardiac SARS-CoV-2 infection (alongside systemic infections) occurred more often in the presence of cardiac hypertrophy. Cardiac SARS-CoV-2 infection was associated with a more aggressive course of the disease and impaired survival compared to patients without cardiac SARS-CoV-2 manifestation.

To further dissect possible underlying mechanisms, we performed immunofluorescence staining of the hearts for ACE2. We found lowered ACE2 and increased cytokine expression levels in hearts with a high viral load. This finding aligns with other reports: By hijacking the ACE2 receptor to infect and injure the cell, SARS-CoV-2 downregulates ACE2 and thereby reduces its physiologic effects^24,26^. Severe downregulation of ACE2 increases pro-inflammatory Ang II signaling and loss of cardioprotective Ang 1–7 effects. This might further stoke a fierce cytokine storm, further aggravating cardiac injury^11,27–29^.

Therapeutic use of the ACE2 receptor is currently investigated: Treatment with a soluble form of ACE2 might impede the cellular entry of SARS-CoV-2 and hence viral spread and protection of cells from subsequent injury^6,28,30^. Other therapeutic interventions might be attenuating the cytokine storm to prevent myocardial injury. Whether one of these strategies might be successful in preventing the deleterious effects of cardiac SARS-CoV-2 infection remains to be elucidated in ongoing trials.

In conclusion, we provide evidence for a correlation between structural heart disease, altered ACE2 receptor expression, and cardiac SARS-CoV-2 infection. Consequently, Structural heart disease may be considered a distinct risk factor for a severe clinical course after infection with SARS-CoV-2.

## Supplementary information

Supplementary Data 1

Supplementary Data 2

Supplementary Data 3

Supplementary Data 4

Supplementary Data 5

Description of Additional Supplementary Files

Reporting Summary

## Data Availability

All source data for figures in the main manuscript are contained in Supplementary Data 1–5. Additional datasets are available upon direct request to corresponding authors. Requests to access additional datasets will undergo internal review and release pending necessary data or material transfer agreements.
