# Supplementary material for: Correlation between structural heart disease and cardiac SARS-CoV-2 manifestations
Source: Commun Med (Lond). 2022 Nov 11;2:142. doi: 10.1038/s43856-022-00204-6 (PMC9651888; doi:10.1038/s43856-022-00204-6)
Supplement: Supplementary file 6 — Description of Additional Supplementary Files [file 43856_2022_204_MOESM6_ESM.pdf]

Description of Additional Supplementary Files

File Name: Supplementary Data 1
Description: Source data including counts of outpatients and inpatients with or without structural heart disease obtained from the CovILD cohort.

File Name: Supplementary Data 2
Description: Source data including counts of COVID-19 fatalities, either with SARS-CoV-2 cardiac involvement or not, with or without structural heart disease.

File Name: Supplementary Data 3
Description: Source data including automatically generated intensity counts (arbitrary units) in immunofluorescence staining for ACE2 in myocardial tissues with or without cardiac SARS-CoV2 involvement.

File Name: Supplementary Data 4
Description: Source data including relative RNA expression of INFa, IL6, ACE2 and TNFa as well as relative SARS-CoV-2 viral load (spike protein) in myocardial tissue sections with cardiac SARS-CoV2 involvement.

File Name: Supplementary Data 5
Description: Source data including survival of patients who died subsequently to a COVID-19 infection with or without cardiac SARS-CoV2 involvement.
