# Supplementary material for: Correlation between structural heart disease and cardiac SARS-CoV-2 manifestations
Source: Commun Med (Lond). 2022 Nov 11;2:142. doi: 10.1038/s43856-022-00204-6 (PMC9651888; doi:10.1038/s43856-022-00204-6)
Supplement: Supplementary file 7 — Reporting Summary [file 43856_2022_204_MOESM7_ESM.pdf]

nature research

Corresponding author(s): Can Gollmann-Tepeköylü
Anichstr.35
Department of Cardiac Surgery, Medical University of Innsbruck, Austria
E: can.gt@i-med.ac.at
T: +43 512 504 80466

Last updated by author(s): Oct 12, 2022

Reporting Summary

Nature Research wishes to improve the reproducibility of the work that we publish. This form provides structure for consistency and transparency in reporting. For further information on Nature Research policies, see our Editorial Policies and the Editorial Policy Checklist.

Statistics

For all statistical analyses, confirm that the following items are present in the figure legend, table legend, main text, or Methods section.

n/a	Confirmed	
☐	☒	The exact sample size (*n*) for each experimental group/condition, given as a discrete number and unit of measurement
☐	☒	A statement on whether measurements were taken from distinct samples or whether the same sample was measured repeatedly
☐	☒	The statistical test(s) used AND whether they are one- or two-sided<br>*Only common tests should be described solely by name; describe more complex techniques in the Methods section.*
☐	☒	A description of all covariates tested
☐	☒	A description of any assumptions or corrections, such as tests of normality and adjustment for multiple comparisons
☐	☒	A full description of the statistical parameters including central tendency (e.g. means) or other basic estimates (e.g. regression coefficient) AND variation (e.g. standard deviation) or associated estimates of uncertainty (e.g. confidence intervals)
☐	☒	For null hypothesis testing, the test statistic (e.g. *F*, *t*, *r*) with confidence intervals, effect sizes, degrees of freedom and *P* value noted<br>*Give P values as exact values whenever suitable.*
☒	☐	For Bayesian analysis, information on the choice of priors and Markov chain Monte Carlo settings
☒	☐	For hierarchical and complex designs, identification of the appropriate level for tests and full reporting of outcomes
☒	☐	Estimates of effect sizes (e.g. Cohen's *d*, Pearson's *r*), indicating how they were calculated

Our web collection on statistics for biologists contains articles on many of the points above.

Software and code

Policy information about availability of computer code

Data collection: Data documentation and statistical analysis were performed using SPSS 24.0 (IBM Corp.) and RStudio Version 1.4 (RSudio Team, Boston, USA).

Data analysis: Data documentation and statistical analysis were performed using SPSS 24.0 (IBM Corp.) and RStudio Version 1.4 (RSudio Team, Boston, USA).

For manuscripts utilizing custom algorithms or software that are central to the research but not yet described in published literature, software must be made available to editors and reviewers. We strongly encourage code deposition in a community repository (e.g. GitHub). See the Nature Research guidelines for submitting code & software for further information.

Data

Policy information about availability of data

All manuscripts must include a data availability statement. This statement should provide the following information, where applicable:
- Accession codes, unique identifiers, or web links for publicly available datasets
- A list of figures that have associated raw data
- A description of any restrictions on data availability

All source data for figures in the main manuscript are contained in Supplementary Data 1 – 5. Additional datasets are available upon direct request to corresponding authors. Requests to access additional datasets will undergo internal review and release pending necessary data or material transfer agreements.

Field-specific reporting

Please select the one below that is the best fit for your research. If you are not sure, read the appropriate sections before making your selection.

- [x] Life sciences
- [ ] Behavioural & social sciences
- [ ] Ecological, evolutionary & environmental sciences

For a reference copy of the document with all sections, see nature.com/documents/nr-reporting-summary-flat.pdf

Life sciences study design

All studies must disclose on these points even when the disclosure is negative.

Sample size	Sample size was determined based on similar studies in this field.
Data exclusions	All data were reported except for the CovILD cohort: Only data relevant to this study were obtained from the CovILD database.
Replication	All stainings and confocal images presented were imaged and analyzed at least twice for a single data point.
Randomization	No formal randomization method was used (not applicable, observational study).
Blinding	Investigators were not blinded to group allocation (not applicable, observational study).

Reporting for specific materials, systems and methods

We require information from authors about some types of materials, experimental systems and methods used in many studies. Here, indicate whether each material, system or method listed is relevant to your study. If you are not sure if a list item applies to your research, read the appropriate section before selecting a response.

Materials & experimental systems

n/a	Involved in the study	
☐	☒	Antibodies
☒	☐	Eukaryotic cell lines
☒	☐	Palaeontology and archaeology
☒	☐	Animals and other organisms
☐	☒	Human research participants
☐	☒	Clinical data
☒	☐	Dual use research of concern

Methods

n/a	Involved in the study	
☒	☐	ChIP-seq
☒	☐	Flow cytometry
☒	☐	MRI-based neuroimaging

Antibodies

Antibodies used	All antibodies used are described in detail in the Materials and Patients section.
Validation	All antibodies have been purchased and are listed in the Materials and Patients section.

Human research participants

Policy information about studies involving human research participants

Population characteristics

Study Population
COVID-19 patients discharged from hospital or outpatients referred to our Outpatient Department of Pneumology at the University Hospital of Innsbruck because of persistent respiratory symptoms in recovery phase were followed up. Diagnosis of COVID-19 was ensured by nasopharyngeal and oropharyngeal swabs.

Inclusion Criteria:
Female and male patients ≥ 18 years.
Confirmed infection with SARS-CoV-2 according to the definition of the Austrian Federal Ministry of Social Affairs, Health, Care and Consumer Protection
Signed and dated declaration of consent by the patient according to ICH-GCP Guidelines.

Exclusion Criteria:
Female and male patients < 18 years
Pregnancy
Dementia

	Declaration of consent by the patient according to ICH-GCP Guidelines not signed<br>Incapacitated patients
Recruitment	All population characteristics are described in detail in the Materials and Patients section.
Ethics oversight	Ethical permission for the use of human material has been obtained from the Ethics Committee of Northwestern and Central Switzerland ID 2020-00629 and the Ethics Committee of the Medical University Innsbruck EK Nr: 1103/2020

Note that full information on the approval of the study protocol must also be provided in the manuscript.

Clinical data

Policy information about clinical studies

All manuscripts should comply with the ICMJE guidelines for publication of clinical research and a completed CONSORT checklist must be included with all submissions.

Clinical trial registration	ClinicalTrials.gov Identifier: NCT04416100
Study protocol	Not applicable (observational study).
Data collection	CovILD Cohort Patients were enrolled between April 29th, 2020 and November 11th, 2020 at the Department of Internal Medicine II, Medical University of Innsbruck (Austria) with two additional study sites: St. Vinzenz Hospital Zams and Rehabilitation Facility Münster (both located in Austria).<br><br>Autopsies of twenty-three COVID-19 fatalities, diagnosed as per ante-mortem nasopharyngeal swab, were performed from March to June 2020 at the the Institutes of Pathology of the University Hospital of Basel (n = 12) and the Cantonal Hospital Baselland, Liestal (n = 11).
Outcomes	Not applicable (observational study).
